# Medical Opinion and Movement

**Published:** 1911-07-15

**Authors:** 


### Medical Opinion and Movement.

### Whooping-cough and Vaccination.

Dr. Mehnert, of Jamestown, draws attention to the fact that, according to his experience, vaccination exercises a most beneficial effect upon the course of an attack of whooping-cough. For some years past he has utilised this remedy in infants of three to nine months old afflicted with an attack of whooping-cough, and has always obtained satisfactory results. As soon as the diagnosis of whooping-cough is established, or even earlier, during the catarrhal stage, he vaccinates the baby—provided, of course, that it has not already been vaccinated—and as the pustules develop the coughing fits diminish and eventually completely disappear at the end of a fortnight at the most. Should the case be in an advanced stage, with signs of cerebral hyperæmia, he advises bleeding in conjunction with the vaccination. Whooping-cough is especially dangerous in young infants, the complications of this disease being more frequent and the more serious the younger the child. Moreover, the most effective drugs against the disease, such as bromides, cannot be used in efficacious doses in young infants, so that should further observations confirm the experience of Dr. Mehnert in regard to the efficacy of vaccination against whooping-cough, this method of treatment should prove a valuable weapon against the disease in such cases.

### Joint Infections.

In a contribution to the *North-West Medicine*, Dr. Clarence L. Roley describes a comparatively new method of treatment of joint infections by means of aspiration and injections of a 2 per cent. formalin solution in glycerine. Early aspiration of accumulations of fluid under pressure in all types of arthritis tends to prevent ankylosis. The aspiration and injection of the formalin in glycerine should be followed by extension with weight and pulley to keep the ends of the bones separated and immobilise the parts in the best position, should ankylosis follow. Ten drops of formalin are added to each ounce of glycerine. The mixture should be kept in a sterile vessel and should not be more than twenty-four hours old when used. The skin should be painted with iodine. With the patient anæsthetised, the aspirating needle is inserted into the joint. Free motion of the needle indicates that the cavity of the joint has been entered. The fluid is aspirated and the formalin solution injected until the joint is completely filled. Equal distribution of the solution may be secured by massage and by movement of the joint. The extension apparatus is then applied. A diffuse hyperæmia occurs on the second day, which may be controlled by an ice-bag. On the fourth day the swelling begins to subside. The extension apparatus may now be removed and the joint massaged. Swelling, pain on passive movement, and inflammation are the criteria for subsequent injections. The interval is usually ten to fourteen days. Two injections are often sufficient, but five may be necessary. The best results are obtained in acute or subacute inflammations. Excellent results have been obtained by the author in cases of tuberculous synovitis. Osteomyelitic cavities may be similarly treated with glycero-gelatine plugs of 1 or 2 per cent. formalin.

### Acute Poliomyelitis.

At a meeting of the Académie des Sciences, Dr. Landsteiner gave an account of some interesting research work he had carried out in conjunction with others in a case of acute poliomyelitis. The patient was a child aged two years, admitted into hospital for pleurisy. A month later the patient developed an attack of acute tonsillitis, with fever and vomiting. Three days later paralysis of the lower limbs, left arm, and muscles of the back and neck occurred, and the child succumbed in the night in consequence of paralysis of the respiratory centres. At the autopsy typical lesions of acute poliomyelitis were found and hypertrophy of the tonsils and deposits in the lacunæ. Emulsions in normal saline were made with portions of the organs and injected into monkeys, in doses of 0.5 c.c. into the brain, and 1.75 c.c. into the peritoneum. In two monkeys inoculated, one with spinal cord emulsion and the other with an emulsion of the tonsil, typical and intense lesions of acute poliomyelitis were found on autopsy. One monkey inoculated with pharynx emulsion developed almost complete paralysis, but recovered. Other monkeys inoculated with emulsions of cervical glands, spleen, salivary glands, and mesenteric glands survived without developing any paralysis. The chief point of interest lies in the fact that the attack commenced with symptoms of acute tonsillitis and that the virus was detected by these inoculations in the tonsils and pharynx, so that the infecting microbe of the poliomyelitis appears to have found entry into the system by means of the tonsils and pharyngeal mucous membranes. The author points out that in certain epidemics the disease is often ushered in by anginal signs and in others by different initial symptoms, such as digestive troubles, so that it seems probable that there are many portals of invasion for the microbe of acute epidemic poliomyelitis.

### A Post-mortem Phenomenon.

In the *Deutsche Medizinische Wochenschrift*, Dr. H. E. Hering calls attention to a post-mortem phenomenon which he has observed on auscultation of the chest immediately after death and which does not appear to have been described hitherto. He was attending a patient at the time of death, and when the last respiratory movements had passed away he applied the stethoscope at the cardiac apex. There was no longer any heart-beat to be heard, but he was surprised to hear a slight continuous murmur. On applying the stethoscope to the lower margin of the sternum he observed the same murmur. The phenomenon was confirmed by his two assistants. The murmur was not rhythmic but continuous, and ceased at the end of one or two minutes. It was soft and appeared to be produced immediately under the point auscultated. A little later one of his assistants observed the same murmur in a similar case, but this time it only lasted about half a minute. In regard to the origin of this bruit, the first idea was that it was produced by a rigor of the heart, but on further reflection it seemed hardly probable that the dissociated contraction of some cardiac muscular fibres would produce such a bruit. According to the author, the most plausible hypothesis would appear to be that the bruit is of vascular origin, and is produced by the blood continuing to flow from the arteries towards the veins for a short time after the heart has ceased to beat. The fact that this occurs has already been shown in the case of animals. Dr. Hering proceeds to show that such a movement of the blood might well produce a bruit such as has been observed by him. He suggests that the same phenomenon takes place during life, but the heart sounds prevent it from being heard.

### Eclampsia.

In Dr. Hastings Tweedy's annual report on the work of the Rotunda Hospital he lays considerable stress on certain points in the management of eclampsia cases, to which he attributes a good deal of the success with which these patients have been treated at the Dublin clinic of late years. Death should not in any case occur during the eclamptic convulsion, he says; for when it does it is really due to falling back of the tongue and spasm of the glottis induced by a collection of mucus in the throat. To avoid this he advises that the right lateral posture should be maintained; but if cyanosis becomes marked he turns the patient over into the prone position, with the head over the end of the bed. Artificial respiration and oxygen are two means of restoration of such patients which should always be given a trial. His other point is in relation to food. Dr. Tweedy believes that food poisoning accounts for eclamptic seizures, and that during the convulsive stages absolute starvation should be the rule. He has the stomach washed out as soon as fits begin, and in many cases recovers thus surprising quantities of undigested food. Heart failure will more certainly result from toxæmia than from inanition, is his concluding remark.

### Embolism and the Trendelenburg Posture.

In a paper abstracted for the *Medical Review*, Zweifel is confident that the Trendelenburg posture is certainly a disposing factor in the production of embolism. On a careful analysis of his operative work since 1887 he was surprised to find that his operation mortality, which decreased steadily down to 1898, has risen since that date. This was the year when he adopted the Trendelenburg posture as a routine measure in abdominal operations; and on investigating his figures he concluded that the increase in mortality is accounted for by the greater frequency of post-operative embolism. On casting about for the explanation of this frequency of embolism, it occurred to him that possibly the tight fastening of the legs with the knees well flexed might account for clotting or impeded circulation in the veins of the leg; so he took measures to counteract such a tendency. He now has the knees extended in horizontal frames, and not at all tightly strapped. The shoulders, too, are propped up; and, in fact, Trendelenburg's position is avoided except when it is absolutely necessary. These measures have been followed by a marked reduction in the incidence of pulmonary embolism in his wards. The figures quoted do not seem to bear out this view entirely, and there may possibly be other explanations of the undoubtedly high death-rate which this author is courageous enough to publish.

### Scurvy.

Dr. Hewetson, who is medical officer to a large colliery in Rhodesia, contributes to the *Transvaal Medical Journal* his views on the ætiology of scurvy, a disease which is rampant among the natives in the former colony. His conclusions are directly counter to most of the commonly accepted hypotheses; and, as he has probably more personal experience of the disease than anyone in this country can have, they are worthy of close attention. So impressed is he with this divergence of view that he finally expresses a faint doubt as to whether the disease he has to treat is "true scurvy," though it certainly coincides with the ordinary text-book description of the latter. Scurvy, he says, is a disease which runs a variable but more or less definite course; it increases in virulence during its special seasonal incidence (in Rhodesia), as many other epidemic diseases do. He has been forced to discard the dietetic theory altogether, and to believe that scurvy is a specific infective disease due to a spirochæte, which in most cases, but not all, first invades the gums. Not once, but hundreds of times, and so constantly that he now regards it as normal, he has seen acute gingivitis appear immediately among the "boys" as soon as brushing and close supervision were relaxed. He suggests that scurvy should now be called acute toxic gingivitis.

### Iodoform in Phthisis.

The intravenous injection of etherial solution of iodoform has been practised by Dr. T. W. Dewar for a number of years, and various papers have been published by him on the subject. In the *Glasgow Medical Journal* he returns to the attack; his extended experience confirms the high opinion he had previously of this line of treatment, and he enters into a comparative discussion of its advantages over sanatorium and tuberculin treatment. In early cases the author claims that complete arrest of the disease is in most cases attained, without interruption of the patient's wage-earning power and without necessity for special diet or other expensive extras. In three or four months he undertakes to make such a patient quite well. In more advanced cases, with distinct breaking-down of lung tissue, loss of flesh, fever, sweating, and so on, the treatment requires to be carried out for a year, and rest, with extra diet, will also be necessary. With tuberculin his experiences have been unfavourable, and he has both theoretical and practical objections to urge against this treatment. How exactly the benefit of iodoform injections accrues he does not do more than speculate. The dosage he is accustomed to employ is ¼ to 1 grain of iodoform three times a week; judgment and care are required not to overdose the patient. In many thousands of injections Dr. Dewar has never caused embolism or hæmoptysis; and with his improved technique he never now gets local clotting at the site of injection.

### High-Frequency Currents for Ozæna.

A recent number of the *Archives d'électricité Médicale* contains a paper by Torres Carreras, in which he describes his treatment of ozæna by high-frequency currents. He commences by making the patient irrigate the nose twice daily until all the crusts have been removed. Then he places a metal electrode on the back of the neck, and introduces the other, the active glass one, into the patient's nose. He makes use of a current of moderate intensity, each sitting lasting from two to five minutes, and being repeated every second or third day. So far nine patients have been treated in this way, and in every case a cure has been effected. The average number of sittings necessary to bring about a cure is a dozen. He explains the good effects of the current by its trophic effect on the nasal mucous membrane, by its stimulating action on the system in general, and by the bactericidal action of the ozone formed round the electrode. He advises that all cases should be first treated by his method. If by chance it should fail, recourse could then be made to injections of solid paraffin fusible at 45° C., which would certainly give better results in the case of a mucous membrane regenerated by high-frequency currents than in cases not so treated.

### Trypanosomiasis and the Rat-flea.

A paper on the mechanism of transmission of *Trypanosoma Lewisi* from rat to rat by the rat-flea, recently appeared in the *British Medical Journal* from the pen of C. Strickland, assistant in the Quick Laboratory, Cambridge. With a view to determining whether infection is the direct result of the flea biting the rat or in what other manner the flea infects the rat, the author made use of batches of ten infected fleas for each rat. Batches of fleas previously starved for three days were placed on rats for half an hour, and then withdrawn. Of nine rats none became infected. Other batches of fleas were then killed and mingled with bread-sop and given to rats to eat. Of nine rats, six became infected. Other batches of fleas were placed on rats and allowed to remain as in nature. Of seven rats two became infected. The author draws the following conclusions from his experiments: 1. Infection of rats with *T. Lewisi* is caused by the rats eating infective fleas. 2. It is not caused by contamination of the rats during a short time, nor by their being bitten by the fleas. 3. Many more fleas are infective than cause infection in nature. 4. The infective form of trypanosome, which is probably the "small trypanosome" of Swellengrebel and Strickland, must reach the blood through the gut-wall. 5. It is possible that other trypanosomes may be transmitted by even non-blood-sucking creatures. In that the *T. Lewisi* is akin to the trypanosome of sleeping-sickness, these experiments are important.

### A New Way of Preparing Hæmolytic Sera.

At a recent meeting of the Paris Society of Biology, M. Jacobson read a paper on the absorption of red blood corpuscles by the rectal mucous membrane. This observer introduced every other day 5 c.c. of sheep's blood into the rectum of a rabbit. He was able at the end of three weeks to show that the serum of this rabbit contained a hæmolysin which was very active in the presence of the red blood corpuscles of the sheep. The rectal mucous membrane had evidently been traversed by the red blood corpuscles which, playing their part as antigen, had brought about the formation of an antibody similar to that produced by direct injection. This experiment furnishes a new method of preparing a hæmolytic serum, and, moreover, one free from dangers of anaphylaxis.

### Pneumonia and Rheumatism.

Dr. T. G. McConkey asks in the *Medical Record* the question—Are pneumonia and rheumatism specific infections? and proceeds to argue that they are not. He recapitulates his argument thus: Pneumonia and rheumatism are merely symptom complexes dependent upon and secondary to tuberculous vegetations or lesions on the pleura, pericardium, endocardium, or periarticular tissues. The streptococcus usually present in the mouth and nasal cavities is the most frequent of these secondary invaders; but staphylococci, Friedlander's bacillus, and the specific micro-organisms of typhoid, diphtheria, gonorrhœa, etc., may also be secondary invaders as well. Whether the symptom complex called pneumonia or that called rheumatism occurs depends upon the accident of the location of this damage of tuberculous causation. If situated on the pleura, pneumonia is the diagnosis, provided consolidation occurs; otherwise the result is called pleurisy. If situated in the joints, alone or in connection with the endo- or peri-cardium, rheumatism is diagnosed. If situated on the endocardium alone, and not followed by consolidation of the lung, it is called endocarditis. These novel and unorthodox views are supported with ingenuity; but many important points are overlooked or omitted, and it cannot be said that the author's presentation of his case carries conviction with it.

